# Molecules, morphometrics and new fossils provide an integrated view of the evolutionary history of Rhinopomatidae (Mammalia: Chiroptera)

**DOI:** 10.1186/1471-2148-7-165

**Published:** 2007-09-14

**Authors:** Pavel Hulva, Ivan Horáček, Petr Benda

**Affiliations:** 1 Department of Zoology, Charles University, Viničná 7, CZ-128 44 Praha 2, Czech Republic; 2 Department of Zoology, National Museum (Natural History), Václavské námĕstí 68, CZ-115 79 Praha 1, Czech Republic

## Abstract

**Background:**

The Rhinopomatidae, traditionally considered to be one of the most ancient chiropteran clades, remains one of the least known groups of Rhinolophoidea. No relevant fossil record is available for this family. Whereas there have been extensive radiations in related families Rhinolophidae and Hipposideridae, there are only a few species in the Rhinopomatidae and their phylogenetic relationship and status are not fully understood.

**Results:**

Here we present (a) a phylogenetic analysis based on a partial cytochrome *b *sequence, (b) new fossils from the Upper Miocene site Elaiochoria 2 (Chalkidiki, Greece), which represents the first appearance datum of the family based on the fossil record, and (c) discussion of the phylogeographic patterns in both molecular and morphological traits. We found deep divergences in the *Rhinopoma hardwickii *lineage, suggesting that the allopatric populations in (i) Iran and (ii) North Africa and the Middle East should have separate species status. The latter species (*R. cystops*) exhibits a shallow pattern of isolation by distance (separating the Middle East and the African populations) that contrasts with the pattern of geographic variation in the morphometrical traits. A deep genetic gap was also found in *Rhinopoma muscatellum *(Iran vs. Yemen). We found only minute genetic distance between *R. microphyllum *from the Levant and India, which fails to support the sub/species distinctness of the Indian form (*R. microphyllum kinneari*).

**Conclusion:**

The mtDNA survey provided phylogenetic tree of the family Rhinopomatidae for the first time and revealed an unexpected diversification of the group both within *R. hardwickii *and *R. muscatellum *morphospecies. The paleobiogeographic scenario compiled in respect to molecular clock data suggests that the family originated in the region south of the Eocene Western Tethyan seaway or in India, and extended its range during the Early Miocene. The fossil record suggests a Miocene spread into the Mediterranean region, followed by a post-Miocene retreat. Morphological analysis compared with genetic data indicates considerable phenotypic plasticity in this group.

## Background

The mammalian order Chiroptera serves as an excellent example of how molecular phylogenetics has influenced the taxonomy of a seemingly well resolved group. Genetic data invalidated the traditional subdivision of bats into suborders Megachiroptera and Microchiroptera when Teeling *et al*. [1-3] provided molecular evidence supporting sister position of one clade of microbats, Rhinolophoidea, with Megachiroptera. For that reason, the actual content of the Rhinolophoidea and phylogenetic structure of that clade became a matter of considerable interest. In the traditional view [4], Rhinolophoidea included Rhinolophidae, Hipposideridae, Megadermatidae, and Nycteridae. Molecular evidence [2,5] has suggested that Nycteridae is not a member of that clade, but a sister group to Emballonuridae, whereas molecular data have brought two previously unassociated groups, Rhinopomatidae and Craseonycteridae, traditionally arranged together with Emballonuridae in the superfamily Emballonuroidea, into the Rhinolophoidea. The molecular taxonomy of Craseonycteridae has also been dealt with briefly [5], but Rhinopomatidae has remained one of the few mammalian families not re-examined from the perspective of molecular genetics (except for the data suggesting its position among Rhinolophoidea, close to Craseonycteridae and Megadermatidae [3,6,7].

The lack of genetic data is particularly frustrating because Rhinopomatidae is, for several reasons, the most enigmatic group of extant bats. It is a monotypic family (composed of a single genus, *Rhinopoma *Geoffroy, 1813) with an exceptionally large geographic range covering a considerable part of tropical subsaharan Africa and most of the southern Mediterranean, Middle East, and southern Asia (from Morocco, Senegal and Kenya, through Arabia and the Middle East, to India, Thailand and Sunda Archipelago) [4,8,9]. Among monotypic families of mammals, only Rhinolophidae, Equidae and Manidae occupy such extensive geographic ranges. In contrast to rich data on the history and relationships of the other two families, no such information is available for Rhinopomatidae: there is no fossil record of the family (except for one occurrence in the Late Pleistocene of Israel [10]) and the relationship of the family to other bats has traditionally been unresolved. Rhinopomatidae bear a unique set of morphological plesiomorphies for which they were often regarded as the most primitive group of Microchiroptera close to the common ancestor of microbats and megabats [4,11-14]. After recent shaking of the chiropteran tree by molecular phylogenetics, rhinopomatids retained their basal position – in morphological respects they are still the most primitive clade within Rhinolophoidea and, thus, also the most primitive extant clade within the Yinpterochiroptera in the sense of Teeling et al. [3]. For all these reasons, Rhinopomatidae are an extremely attractive subject for a detailed study. Yet, these bats are quite rare throughout their range and recent records from many important regions are simply not available. Consequently, very few authors succeeded in comprehensive investigation of this taxon and its current taxonomy reflects numerous uncertainties about its actual content.

**Figure 1:**
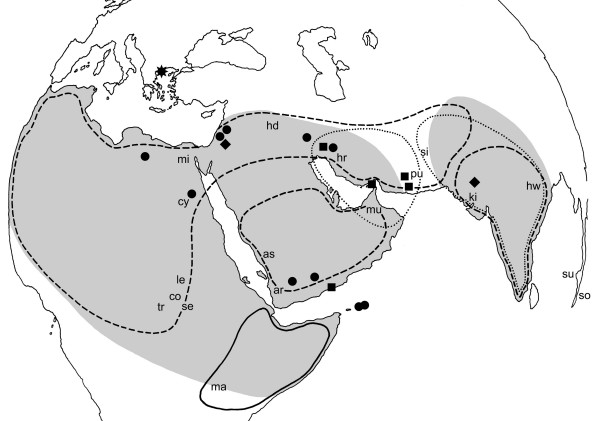
**Extant geographic distributions of species of rhinopomatid bats**. (after [9, 56]): gray grid – *R. hardwickii *lineage, dashed line – *R. microphyllum*, dotted line – *R. muscatellum *lineage, full line – *R. macinnesi*. Localities of the DNA samples used in the present paper (comp. Table 1): dots – *R. hardwickii *lineage, diamonds – *R. microphyllum*, squares – *R. muscatellum *lineage, asterisk – fossil *Rhinopoma *aff. *Hardwickii*. Type localities of individual named taxa: ar – *arabium *Thomas, 1913, as – *asirensis *Nader et Kock, 1982, co – *cordofanicum *Heuglin, 1877, cy – *cystops *Thomas, 1903, hd – *hadithaensis *Khajuria, 1988, hr – *harrisoni *Schlitter et DeBlase, 1974, hw – *hardwickii *Gray, 1831, ki – *kinneari *Wroughton, 1912, le – *lepsianum *Peters, 1859, ma – *macinnesi *Hayman, 1937, mi – *microphyllum *Brünnich, 1782, mu – *muscatellum *Thomas, 1903, pu – *pusillum *Thomas, 1920, se – *sennaariense *Kock, 1969, si – *seianum *Thomas, 1913, so – *sondaicum *Van Cakenberghe et De Vree, 1994, su – *sumatrae *Thomas, 1903, tr – *tropicalis *Kock, 1969.

The present paper provides the first phylogenetic study on the family Rhinopomatidae based on the samples subsequently collected from the regions situated in the centre of the family range, i.e. Levant, Arabia, Iran, India and NE Africa (Figure 1). The results suggest that the taxonomic structure of the group is rather more complicated than commonly expected. In previous taxonomies, the genus *Rhinopoma *was divided into two to seven species and several local forms, for which at least 17 names are available. The detailed taxonomic review [see Additional file 1] demonstrates complicated and often opposing viewpoints and illustrates that attempts to reconstruct the structure of this family on the basis of classical markers alone were seldom persuasive. The most recent and comprehensive revision of the group [9] established a set of diagnostic morphological characters which splits the genus into four separate species sharing a broad range of sympatry. This arrangement has become the standard taxonomy [comp. [9,15]] and it is adopted as the null hypothesis in the present paper (i.e. we refer to *R. hardwickii*, *R. muscatellum *and *R. microphyllum *lineages). The phylogroups revealed by our investigation are distinguished by Roman numerals (I–V), their nomenclatorial assignments summarized in Table 1 and explained in Additional file 1. We also report the first Neogene fossil record of Rhinopomatidae and discuss the history of this unique family of bats.

**Table 1:** Taxonomic structure of Rhinopomatidae: a tabular survey

**Molecular phylogroups**	**Range**	**Taxonomy**	
this paper		**traditional **after Simmons 2005	**proposed **this paper

**I**	Iran	*R. hardwickii hardwickii * Gray, 1831	*R. hardwickii ***ssp. n.**

*	India (*T*: Bengal) to Thailand	*R. hardwickii hardwickii * Gray, 1831	*R. hardwickii hardwickii * Gray, 1831

*	Sunda Archipelago	*R. hardwickii sondaicum *van Cakenberghe & de Vree, 1994	*

*	Sub-Saharan Africa	*R. hardwickii arabium * Thomas, 1913	*

**IIa**	Levant	*R. hardwickii arabium * Thomas, 1913	*R. cystops arabium * Thomas, 1913

**IIb**	W Yemen (*T*)	*R. hardwickii arabium * Thomas, 1913	*R. cystops arabium * Thomas, 1913

**IIb**	Socotra	*R. hardwickii arabium * Thomas, 1913	*R. cystops arabium * Thomas, 1913

**IIc**	Upper Egypt (*T*)	*R. hardwickii cystops * Thomas, 1903	*R. cystops cystops * Thomas, 1903

**IId**	NE Libya	*	*R. cystops cystops * Thomas, 1903

**III**	SW Yemen	*R. muscatellum * Thomas, 1903	*R. ***sp. n. ** (aff. *muscatellum*)

**IV**	SW Iran (Oman *T*)	*R. m. muscatellum * Thomas, 1903	*R. m. muscatellum * Thomas, 1903

*	Pakistan, SW India	*R. m. muscatellum * Thomas, 1903	*

*	Lower Egypt (*T*)	*R. microphyllum microphyllum * (Brünnich, 1782)	*R. m. microphyllum * (Brünnich, 1782)

**V**	Levant	*R. m. microphyllum * (Brünnich, 1782)	*R. m. microphyllum * (Brünnich, 1782)

*	SW Saudi Arabia (*T*)	*R. m. asirensis * Nader & Kock, 1982	*

**V**	India	*R. microphyllum kinneari * Wroughton, 1912	*R. m. microphyllum * (Brünnich, 1782)

*	Thailand, N-Sumatra	*R. microphyllum sumatrae * Thomas, 1903	*

*	Morocco to sub-Saharan Africa	*R. microphyllum * (Bruennich, 1782)	*

*	Kenya (*T*) to Eritrea	*R. macinnesi * Hayman, 1937	*R. macinnesi * Hayman, 1937

+	Elaiochoria, Greece, MN10-13	*	† *Rhinopoma * **sp. n. **(aff. *hardwickii*)

The molecular phylogroups covered by this paper are denoted by Roman numerals (I to V), those not covered but referred to populations of expected taxonomical significance are denoted with an asterisk (*) similarly as absence of a taxonomic opinion. (*T*) refers to a 'topotype' population, + to a fossil taxon.

## Results

### Morphometry

**Figure 2:**
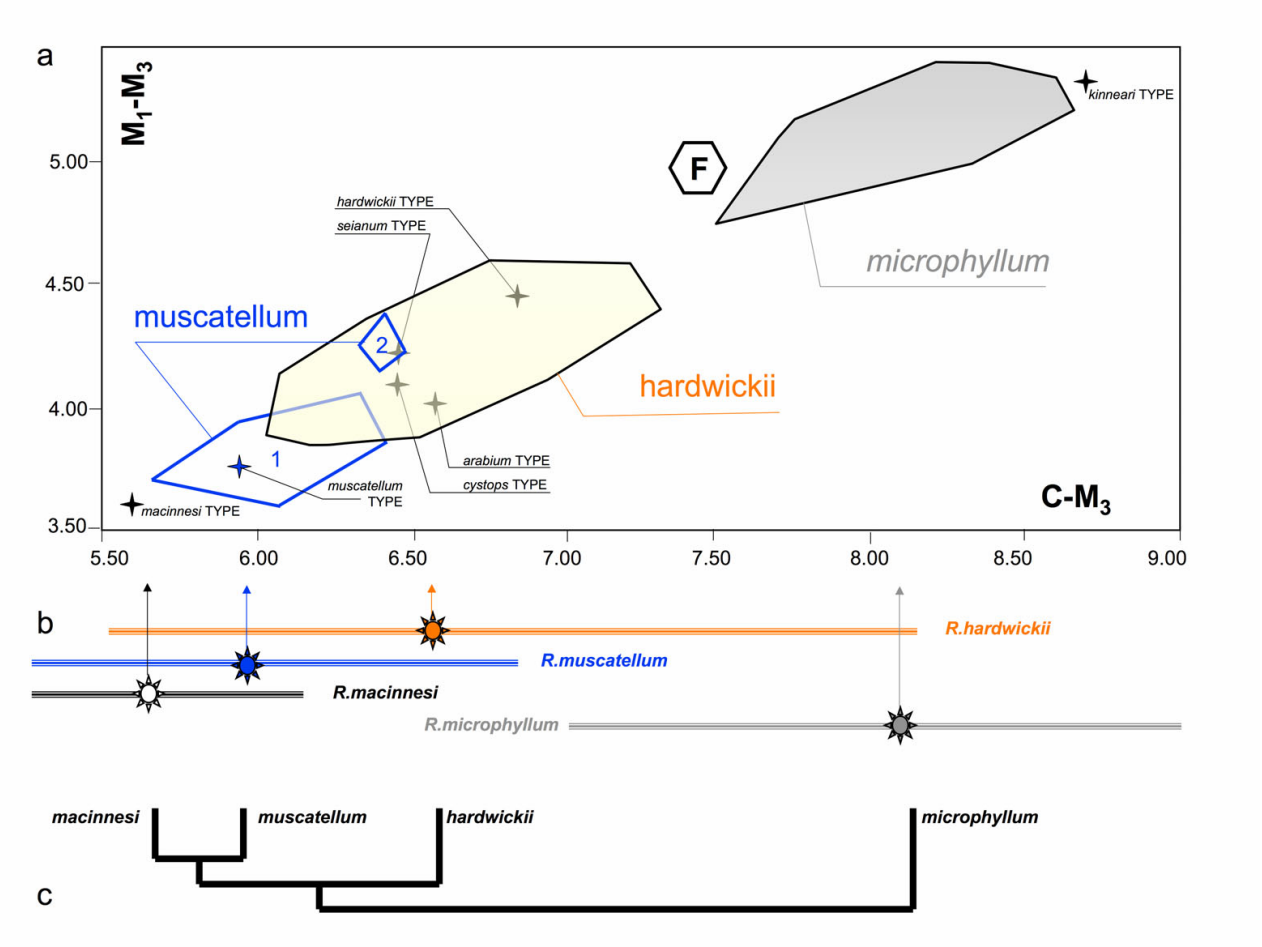
**Morphometric characteristics of Rhinopomatidae**. **a **– scatter plot of CM^3 ^vs. M^1^–M^3 ^in the sample examined in frame of this study (n = 252, for further data see [28, 29]) with position of the respective type specimens: note metric distinctness of the phenotypic forms *hardwickii*, *microphyllum*, *muscatellum *(1 – Iran, 2 – Yemen) and position of the Miocene fossil from Elaiochoria 2, Greece; **b **– mean values and variation span of CM^3 ^in four species of *Rhinopoma *as reported by Van Cakenberghe and De Vree [9], n = 357, 63, 54, 154; **c **– phylogenetic hypothesis suggested by morphometric characters.

Despite using a considerably extended set of morphometrical characters and applying multivariate morphometric analyses (based on 252 specimens, including types of 7 taxa, see [16] for details) our results revealed the same pattern of variation as reported in detail by Van Cakenberghe and De Vree [9], and Benda et al. [16,17]. *Rhinopoma microphyllum*, *R*. *hardwickii*, and *R. muscatellum *are distinct with partial overlap between the latter two (Figure 2). These phenotypic groups (or morphospecies) were found to be internally homogenous in their diagnostic characters, with *R. hardwickii *having the pronounced intraspecific variation, particularly in metrical components of body size [for details see Additional file 1].

### Molecular analyses

We obtained 26 sequences from ingroup taxa of the first 402 bp of the cytochrome *b *gene. Ninety-nine positions were variable, 93 were parsimony informative, and all mutations were base substitutions. The *Rhinopoma *samples analyzed fell into 15 haplotypes. The sequences exhibited a low level of saturation at the first and second codon positions, with a deflection from linearity at third codon positions. We used a GTR + I + G distance correction model with gamma distribution shape parameter = 1.70.

**Figure 3:**
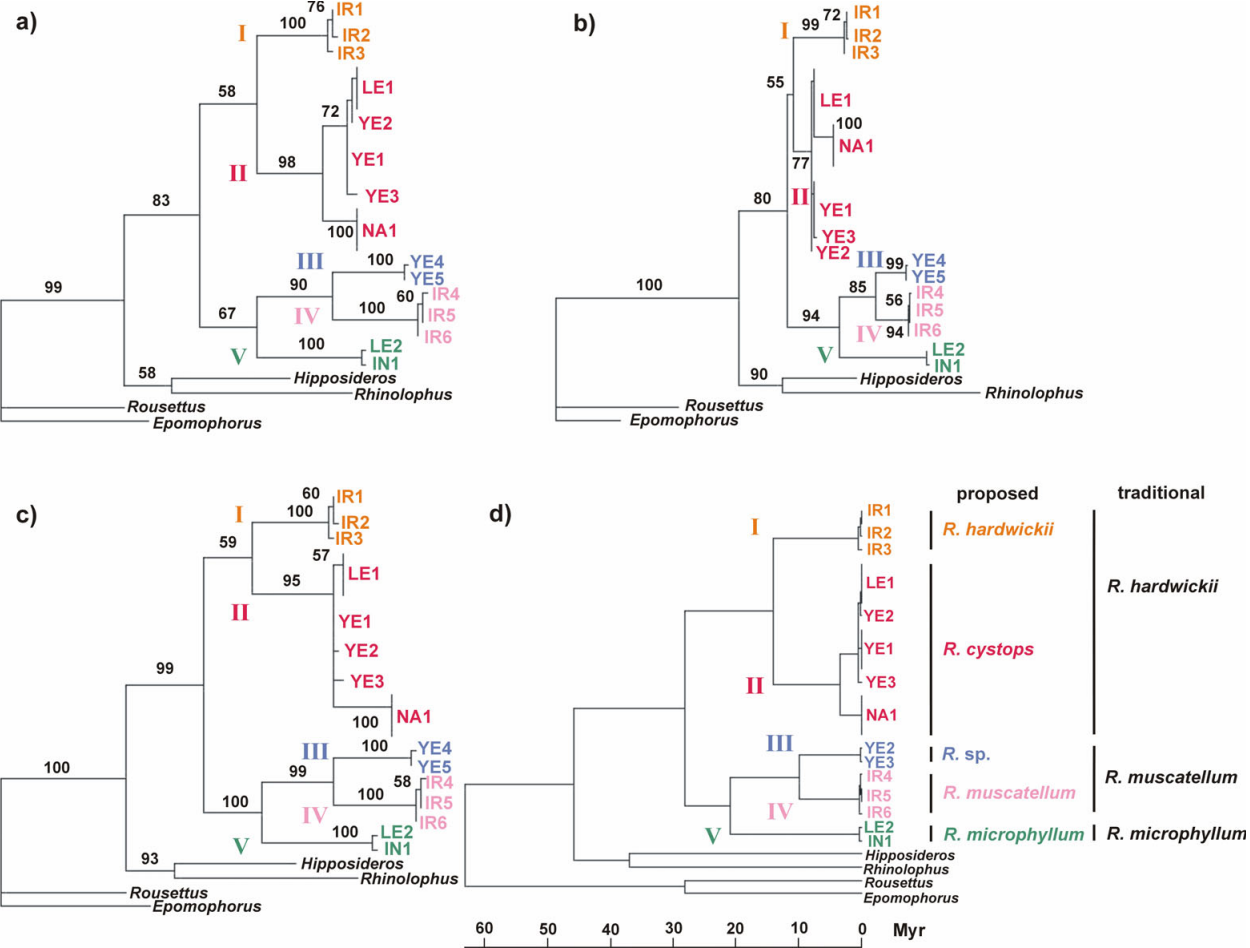
**The topology of the rhinopomatid tree reconstructed from the 402 bp sequence of cytochrome *b *gene**. **(a) **maximum parsimony tree, length = 320 mutations, consistency index excluding uninformative characters = 0.57; retention index = 0.82; rescaled consistency index = 0.50 **(b) **maximum likelihood tree calculated under GTR+I+G model of sequence evolution, R-matrix = (2209.1157, 5744.5737, 1027.7924, 0.0014, 23577.1543, 1.0000), base frequencies = (0.2945, 0.3553, 0.1347, 0.2154) and gamma shape parameter = 1.6997, log*L *= -1867.36 **(c) **Bayesian tree with the same model of sequence evolution as in ML method **(d) **linearized maximum likelihood tree with the same model of sequence evolution as in ML method and molecular clock enforcement, log*L*_clock _= -1884.45. Numbers at the nodes correspond to 1000 replication bootstrap supports/posterior probabilities.

**Figure 4:**
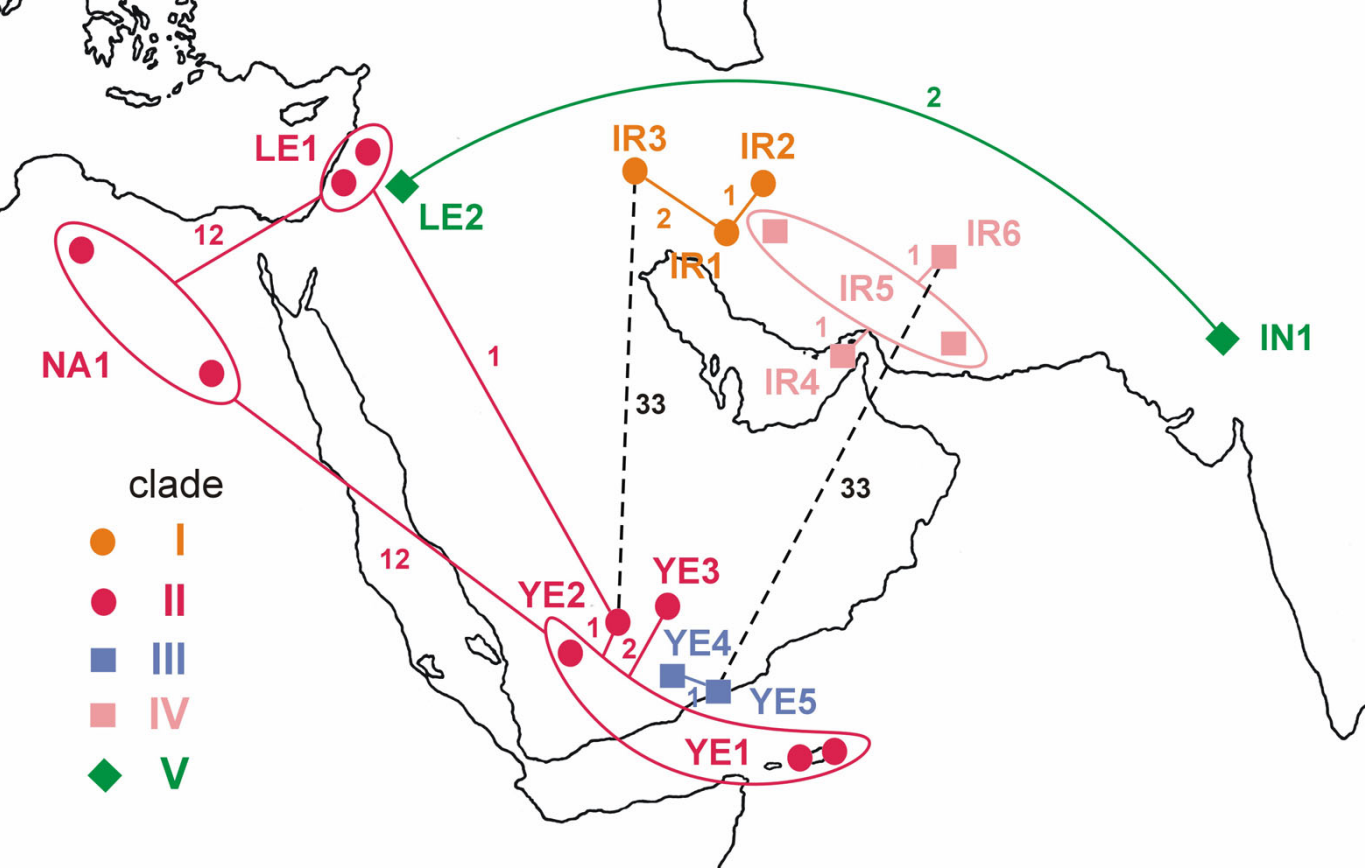
**Geographic arrangements of parsimony networks connecting mtDNA haplotypes of bats from the genus *Rhinopoma *within clades revealed by tree building methods**. Numbers at the branches indicate number of mutational steps. Dashed lines – the branches with minimum number of mutations between clades I and II and clades III and IV. The superimposition of the network in Iran, Yemen and Levant do not match geography exactly due to space limitations.

All tree-building methods resulted in recognition of five clades, highly supported by bootstrap values and posterior probabilities (Figures 3 and 4): (clade I) Iranian haplotypes of *R. hardwickii*; (clade II) Middle Eastern and north African haplotypes of *R. hardwickii*; (clade III) Yemeni haplotypes of *R. muscatellum*; (clade IV) Iranian haplotypes of *R. muscatellum*; and (clade V) haplotypes from the two available specimens of *R. microphyllum*.

The Middle Eastern and north African haplotypes of *R. hardwickii *(clade II) were subdivided into four geographic regions: (a) the Levant (Jordan and Syria); (b) Yemen and Socotra; (c) Upper Egypt; and (d) northern Libya. In contrast to the morphometric data that suggested considerable variation within that clade and support the distinctness of its traditionally recognized subspecies (viz. the large-sized *R. h. arabium *vs. the smaller *R. h. cystops *from the Upper Egypt) the genetic data demonstrated an unexpected homogeneity in clade II. A shallow but distinct divergence (p-distance = 3%) was found between the North African haplotypes (c + d) and those from the Levant and Yemen (a + b). No genetic difference was found between (c) and (d), which represent the most distinct forms of the clade in terms of morphometry (Figs. 2, 3 and 4), and only 0.5% genetic distance was found between (a) and (b), which are the most distant geographically. In contrast to the inner shallow cline of genetic variation, clade II is separated from the Iranian one (clade I) by a relatively deep genetic gap (8–9%). Deep divergence was also found within the *R. muscatellum *lineage. The Yemeni haplotypes (clade III) and the haplotypes identified in Iran (clade IV) differ by a genetic distance of about 8–9%. Although the two available specimens of *R. microphyllum *(clade V) represented very distant geographic regions (Levant vs. India) and the local forms have traditionally been considered to be distinct subspecies or even separate species (*R. microphyllum *and *R. kinneari*), their genetic distance was very small (0.5%).

Interestingly, reconstruction of the topology of deep branches within the family using outgroup rooting resulted in sister positions for *R. microphyllum *and *R. muscatellum *lineages (Figure 3). The molecular clock analysis provided the minimum estimate of radiation of the family, i.e. the separation of the *R. hardwickii *lineage, to be approximately 28.1 Ma. The next step, the splitting of the *R. microphyllum *and *R. muscatellum *lineages, was dated minimally to about 20.9 Ma. The split between clades I and II happened about 14.1 Ma, and the split between clades III and IV about 10.0 Ma.

### Fossil record

We found a rich sample of fossil remains unquestionably belonging to *Rhinopoma *sp. in a lithified infilling of a limestone karst cavity in Elaiochoria (Chalkidiki, Greece). The fauna indicates an early Turolian age, Late Miocene (MN 10–11), some 8–10 Ma. The analysis of the record [see Additional files 2 and 3] shows that: (i) the fossil material contains the all the morphological features that clearly distinguish Rhinopomatidae from other chiropteran families and, moreover, it falls within the variation of the Recent forms of the genus; (ii) the morphology corresponds to the Recent *Rhinopoma hardwickii *in the fine details of the dentition, in the shape of the distal epiphysis of the humerus, and in the shape of the proximal epiphysis of the radius; and (iii) the fossil material shows relatively large size variation and seems to exceed the range of variation in the Recent *R. hardwickii*, both in mean values and in the highest values (comp. Figure 2).

## Discussion

Even with the present analysis, the family Rhinopomatidae remains an enigmatic group whose history, taxonomic content, patterns of variation and phylogenetic relationships are far of being properly comprehended. Nevertheless, the data summarized here substantially improve the scarce information on these subjects. We will discuss them in regard to (a) composition of the group, (b) possible phylogeographic patterns and (c) evolutionary history.

### Composition of Rhinopomatidae

The analysis of the family Rhinopomatidae by Van Cakenberghe and De Vree [9] demonstrated that the genus consists of four species that differ in the shape of the palatal incision, the rostral ridges, the narial swellings, in the relative length of the tail, and in overall body size. The most distinctive in all these characters was *R. microphyllum*, whereas the differences between the remaining forms were less pronounced, exhibiting a broad measure of overlap in most metrical characters. Our analysis (*n *= 252) provided the same picture (Figure 2). All of these data suggest that the major phenotypic divergence within the genus is that between *R. microphyllum *(including *R. m. kinneari*, *R. m. sumatrae *and *R. m. asirensis*) and the remaining forms, which thought to be closely related to a medium-sized species, *R. hardwickii *(Figure 2c), see [18].

Our mtDNA study confirmed the existence of the same three deep lineages recognized as morphospecies by Koopman [4], Hill [8] and Van Cakenberghe and De Vree [9] (another recognized species, *R. macinnesi*, was not included in our comparison). In addition, we found (i) deep divergence within the *R. hardwickii *lineage, (ii) incongruency between genetic and phenotypic phylogeographic patterns in clade II, (iii) deep divergence within the *R. muscatellum *lineage, (iv) a very shallow distance between the samples of *R. microphyllum*, suggesting an unexpected genetic homogeneity of that species. Last but not least, we demonstrated that (v) *R. muscatellum *(including the Yemeni population) is not a sister group of *hardwickii*, but of *microphyllum*. All these results contradict the standard view of the taxonomic structure of the family (Table 1), as well as of its distributional history [e.g. [9,15]] and call for a brief comment.

### Phylogeographic patterns

(i) The genetic divergence found within *R. hardwickii *s.l.(= the *R. hardwickii *clade) splits the corresponding morphospecies into an Iranian clade I (*R. hardwickii *s.s.) and Afro-Arabian clade II (*R. cystops*). While there is a clear genetic continuity between the Levantine and Yemeni populations (e.g. haplotypes LE1, YE2, which are separated by approximately 3,000 km, differ by only 1 mutation step), the much smaller geographic distance between the Levantine and Iranian samples (approx. 1,200 km) is combined with deep genetic dissimilarities (the minimum genetic distance between haplotypes LE1 and IR3 is 34 mutation steps). We expect the divergence between these two groups represents real phylogeographic structure, a break crossing the Middle East from the north-west to the south-east. The boundary might be situated along the southern part of the Zagros Mountains, which represents a significant distribution barrier to many clades [17,19]. Unfortunately, knowledge of the distribution of bats in upper Mesopotamia is too scarce [19] to allow further discussion. Thus, we are unable to answer whether there is continuous distribution of haplotype frequencies with a clinal transition between geographic extremes, whether there are two allopatric ranges separated by a distinct geographic gap, or whether the ranges meet at a distinct zone of parapatry or sympatry. Because of the extent of the genetic dissimilarity, we are rather skeptical about the first alternative. Rather, we expect that clades I and II are entities separated at species level. We propose a separate species status for the two clades as per the genetic species concept [20-22] which sets a cutoff based on empirical data (cytochrome *b *in the order Chiroptera) of about 5% of corrected sequence divergence [e.g. [23,24]]. These two groups have almost double that divergence with 9% corrected divergence.

(ii) Within the clade II, a divergence of about 3% separates African and Asian haplotypes of *R. hardwickii*. Within the African group, our genetic data contradict the groupings proposed by previous studies [see [8,9,17] and [25]], which stress a separate status for the populations of the central Sahara (including that of Upper Egypt). The genetic relatedness of these small bats to the largest form in Libya suggests an unexpected degree of phenotypic plasticity in these bats, apparently driven by temporary local conditions rather than by the genotypic backgrounds of the respective populations. Here, the selection pressures of the extreme conditions of desert habitats may have played a key role. A similar pattern of morphological bimodality has been observed in other desert or semi-desert species of bats [16,25,26], such as *Taphozous nudiventris *Cretzschmar, 1830, *Rhinolophus clivosus *Cretzschmar, 1828, *Asellia tridens *(Geoffroy, 1813), or *Pipistrellus kuhlii *(Kuhl, 1817), and such an explanation could be also invoked with respect to the smaller Arabian form, *Rhinopoma microphyllum asiriensis *Nader and Kock, 1983.

(iii) Our study has revealed that the morphospecies *R. muscatellum *is composed of two distinct clades: clade III in Yemen and clade IV in Iran and, supposedly, in Oman. This split is supported by morphometric differences (Fig. 2). Recent allopatry is more obvious in this case because clades III and IV are geographically isolated by the Arabian Desert. However, geographic positioning of major genetic breaks in *R. hardwickii *and *R. muscatellum *lineages coincides with this division (Fig. 4). With respect to the genetic species concept, it is reasonable to consider species status also for clades III and IV [for taxonomic rearrangements in the *R. muscatellum *lineage, see Additional file 1].

(iv) Considering the relatively deep genetic divergences within the morphospecies *R. hardwickii *and *R. muscatellum *(in the sense of Van Cakenberghe and De Vree [9]), the surprisingly low degree of geographic divergence of mtDNA in *R. microphyllum *calls for a comment. At least two qualities of this species are worth discussing in this connection: (i) its larger body size, and (ii) the well-pronounced seasonality of its life cycle and reproduction, including regular seasonal movements [e.g. [27]]. Both of these factors may contribute to increases in vagility and the rate of gene flow.

(v) The sister status of the *R. microphyllum *and *R. muscatellum *phylogroups contradicts traditional arrangements of the family where *R. hardwickii *and *R. muscatellum *are considered as the most closely related taxa based on similarities in narial morphology and body size (Figure 2c) [18,28]. The morphological polymorphism in genetically uniform populations of *R. cystops *(clade II) and *R. microphyllum *(clade I) does, however, indicate that the body size can undergo rapid rearrangement regardless whether in reaction to environmental conditions or as a character displacement due to interspecific interactions. Worth mentioning in this context is the large body size of the fossil *Rhinopoma *aff. *R. hardwickii*, which clearly exceeds the limits of the Recent *R. hardwickii *to which the fossil form is linked by its morphological characters. All these cases suggest that body size, traditionally applied as a significant character in taxonomy of the genus (because of considerable uniformity in other morphological characters) is controlled by ecological factors rather than by a strict taxon-specific developmental constraint.

### Evolutionary history

The evolutionary history of Rhinopomatidae is a subject of particular interest, one which makes the group one of the most enigmatic clades of chiropterans. In the traditional view, Rhinopomatidae were regarded as the most primitive group of extant bats, the closest to the common ancestor of microbats and megabats [12,14]. Indeed, compared to other families of Yangochiroptera and Yinpterochiroptera, the family Rhinopomatidae exhibits a set of unique plesiomorphies: (i) the trochiter of the humerus (tuberculum minor) is small and does not permit the scapulo-humeral lock found in other bats; (ii) the wing tip index has the lowest value of all Chiroptera; (iii) medial phalanx of the second wing finger is complete and well-ossified; (iv) the last cervical and first throracic vertebrae are free (not fused as in other bats); (v) individual sacral vertebrae have distinct boundaries; (vi) the uropatagium is incomplete; (vii) the calcar is absent; (viii) tail is long and mouse-like, not entirely integrated to the uropatagium; (ix) the premaxillae are not attached to each other or to maxillae; and (x) the premaxillae are developed at the palatal plane only. A few of these characteristics (i, iv, vii) are shared with Craseonycteridae, while the others are unique among both Yangochiroptera and Rhinolophoidea, partly resembling the condition in Pteropodidae (i, iii, iv, v, vi, ix, partly vii, viii, x).

In contrast to the major clades of Yinpterochiroptera [cf. [3,15]], Rhinolophidae (1 genus, ca. 77 species), Hipposideridae (9 genera, ca. 81 species), and Pteropodidae (42 genera, ca 184 species), the family Rhinopomatidae is much less diversified [15]. In that respect it is similar to Megadermatidae (4 genera, 5 species) and Craseonycteridae (1 genus, 1 species), which are the sister clades of Rhinopomatidae according to the recent molecular data [3].

The present paper dates the beginning of radiation of extant clades of Rhinopomatidae (i.e. the separation of the *R. hardwickii *clade), to about 29 Ma in the Oligocene. Nevertheless, the datum is apparently not relevant for the beginning of the family which arose with the earliest divergence of Rhinolophoidea, which molecular clock studies place at 50–55 My [3,7]in the Early Eocene. In contrast to other groups of Rhinolophoidea, whose early divergence is well represented in the fossil record, no such information is available for Rhinopomatidae and Craseonycteridae. In contrast to Creaseonycteriae, Rhinopomatidae occupies quite a large range comparable to that of other rhinolophoid families. At least for that reason, the absence of fossil record is unusual and calls for comment, at least as a background story to the discussion on meaning of the first Neogene record of the family reported in this paper.

Despite the fact that the fossil record of bats is sometimes regarded as being quite a poor [3], it is actually rich enough to enable discussions on major differences in phylogeny and early paleobiogeography of particular chiropteran clades at least in Europe and northern Africa. The remains of bats, including rich and taxonomically diversified assemblages, have been found in more than 130 European and North African sites of the Late Eocene, Oligocene and Early Miocene age [29-31] and current views on the structure of chiropteran fauna and the history of particular clades during that period [32] can be considered relevant and reliable. Among Rhinolophoidea, Hipposideridae and Rhinolophidae are particularly rich in their fossil record and, with a number of divergent clades, they have predominated the fossil assemblages in Europe, Africa, and even Australia since the Late Eocene [32-34]. In contrast, no relevant fossil record is available for Craseonycteridae or (until this paper) for Rhinopomatidae. The situation with Megadermatidae is more intricate. The first evidence of appearance of true *Megaderma *in Europe comes from the Upper Oligocene (MP25 Carrascosa del Campo, Spain [35]; MP29 Saint-Victor-la-Coste, France [36]; MP 29 Herrlingen 9, Germany [37]) and a number of further records are of Miocene and Pliocene age [37]. In contrast to hipposiderids or nycterids, the family is absent from African Oligocene sites (including Fayum or Taquah in Oman [30,38] but appears in the Lower Miocene of Thailand and even in Australia [39]. The Late Eocene to Early Oligocene genus *Necromantis*, often regarded as a megadermatid [31], differs from true megadermatids in several characters (including basisphenoidal pits, a key character of emballonurids, which is invariably absent in rhinolophoids) and most probably does not belong to that stock. The absence of Rhinopomatidae and the late first appearance datum of Megadermatids in the fossil record contrasts with the fact that other groups such as Emballonuridae, Hipposideridae, Rhinolophidae, Molossidae, and Vespertilionidae s.l. are constant components of the western Palaearctic and African fossil record since the late Eocene [31,29,40]. All had already produced a number of subclades during the Oligocene and Early Miocene [32,37,41]. The absence of any rhinopomatids in the fossil record is surprising because these bats differ from all others in a number of conspicuous dental and skeletal specificities by which they are easily distinguishable, even based on a single fragmentary tooth. Moreover, rhinopomatids are cave-dwellers, which predisposes them to be particularly common in the fossil record. Under such conditions their absence in fossil record can be interpreted as a real fact which most probably reflects actual absence of the group in the western Palaearctics prior to the Miocene.

The fact that the phenotype of Rhinopomatidae (similarly as in Craseonycteridae) is composed almost exclusively of the ancestral characters not affected by adaptive rearrangements common in other chiropteran families, in contrast to other chiropteran families [42], suggests that (i) the clade was established at a very early stage of chiropteran radiation (prior to the first appearances of modern families, in the Middle Eocene or earlier), and (ii) that rhinopomatids were relatively little affected by the same adaptive processes that affected other all bat families, which all evolved under constant competitive pressure from other microbat clades. The latter could happen only under conditions of long-term isolation of rhinopomatids from other bats. An analogous case is the extinct clades of Palaeochiropterygidae and Hassianycteridae, which extensively diversified in Central Europe during geographic isolation of that region in the Early and Middle Eocene [43,44]. These endemic groups were radically replaced by modern bat families soon after their invasion during the Late Eocene "grand coupure" [33]. The respective modern clades, Hipposideridae, Rhinolophidae, modern Emballonuridae, Vespertilionidae, Molossidae [comp. [29,31]], arrived either from Africa and or Asia, and their early radiations most probably took place there (comp. also [3] for molecular support to that hypothesis). The complete absence of rhinopomatids in the fossil record and the lack of coevolutionary influence on their phenotype suggest that this group was absent in Europe and probably also in Africa and Asia. Of course, Tanzanycterididae with *Tanzanycteris mannardi *from the Early Lutetian (46 Ma) of Tanzania [45] may ultimately be shown to be closely related to Rhinopomatidae. Unfortunately the characters available in the specimen of *Tanzanycteris *provide only tentative support for such a possibility (e.g. enlarged cochlea, a lack of scapulo-humeral lock which is, common to all other Eocene bats).

The first appearance datum of Megadermatidae s. str. is nearly synchronous with dramatic rearrangements of the European mammalian fauna, with the appearance of the Asiatic elements (e.g. Cricetidae) and a considerable contribution of non-mammalian taxa from the Indian and Indomalaysian provinces such as *Gavialosuchus*, *Tomistoma*, and Varanidae. The spread of these taxa into Europe has been dated to ca. 18 Ma [46]. Recent paleogeographic analyses [47,48] supplement the picture with further data that show continuity between the Mediterranean-Iranian and the eastern Indian-east African marine provinces until the final disappearance of the Western Tethyan seaway in the Early Miocene.

In case of Rhinopomatidae, no such evidence is available. The Late Miocene fossil record reported in this paper is apparently not related to the early history of the clade. Nevertheless, the results of molecular studies provide valuable information. The first dated split within the family (*R. hardwickii *s.l. vs. *R. microphyllum-muscatellum*: 28.1 Ma) shows no phylogeographic signal – both clades are broadly sympatric. Nevertheless, the next cladogenetic events (*R. microphyllum *vs. *R. muscatellum*: 20.9 Ma) have clear phylogeographic correlates. In the later events, the phylogeographic signals are even more pronounced: clades I vs. II (14.1 Ma): Iran vs. Levant to Africa, and clades IV and III (10 Ma): Iran vs. Yemen. According to traditional biogeography [49,50], the region with the largest concentration of taxonomic diversity is the most likely candidate for being the source area of the group in question. In the case of rhinopomatids, the present results would suggest Iran to be such a candidate. At the very least, these results suggest that Iran was an area of paleoendemism that played host for the ancestral clades more than 11 Ma ago. Nevertheless, the terrestrial conditions appeared in the respective region first at the time of Oligocene/Miocene transition [51] and thus the source area of the clades that colonized at in that time was apparently situated in other regions.

**Figure 5:**
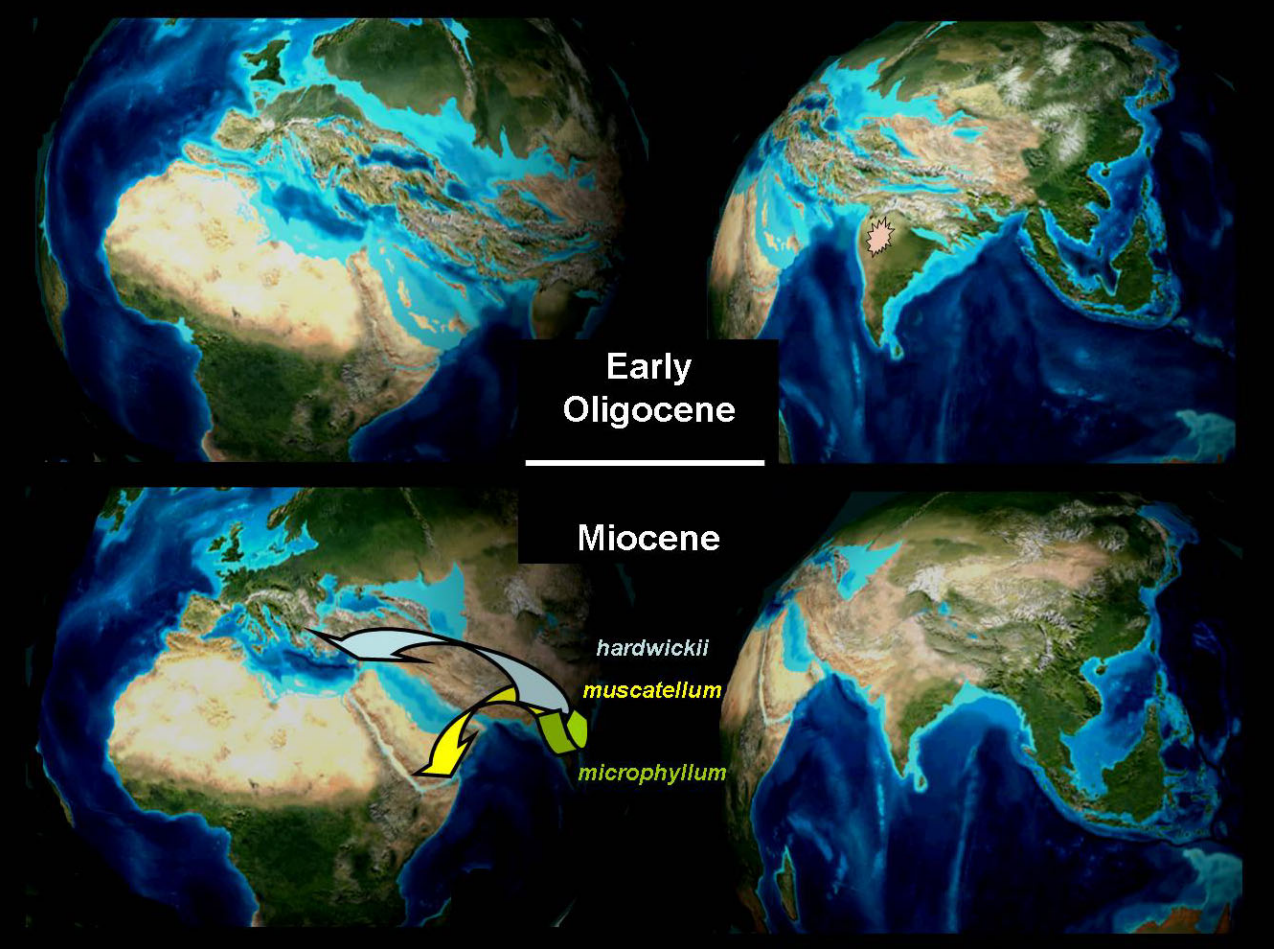
**Palaeogeographic situation of the Indian-Middle East region in the Late Eocene/Lower Oligocene (35 My) and in the Miocene (20 My), with expected expansion pathways of three major lineages (sensu Van Cakenberghe and De Vree [9]) of the family**. The paleogeographic background maps were compiled by Ron Blakey, Department of Geology, Northern Arizona University, Flagstaff [66]. Printed with permission.

Based on the above discussion, we proposed the following biogeographic hypothesis (Figure 5):

(i) Rhinopomatidae originated during the Eocene from the early diversification of rhinolophoid bats that remained isolated from competitive pressure of other chiropteran clades somewhere in the archipelago south of the Western Tethyan seaway or in India.

(ii) The group evolved in isolation until the Oligocene when the marine barrier between the Mediterranean and Indian Tethys provinces disappeared. Endemic adaptation to major chiropteran foraging strategies in ancient rhinolophoids produced clades whose ecomorphological design was much different corresponding foraging specialists on neighbouring continents. Some of those ancient clades survive now: rhinopomatids as aerial foragers, craseonycterids as foragers of small prey in cluttered habitats, and megadermatids as ground gleaners.

(iii) The land between Iran and western India, uplifted during the Oligocene, was subsequently invaded by rhinopomatids and became the key location of their early Neogene radiation. The westward invasion was from the south, then to Arabia (which came in contact with the Iran belt some 20 Ma ago) with the *R. muscatellum *lineage, and perhaps later to the northern part of the Iran and to the Mediterranean with the *R. hardwickii *lineage. The paleobiogeographic analyses of rodents [52] suggest that the respective westward migrations may begun even much earlier – in the late Eocene and early Oligocene via archipelagos south of the Western Tethyan seaway.

(iv) Extension of the paleogeographic and paleoenvironmental rearrangements of the Middle East during the Vallesian and Turolian stage, ca. 11 Ma [compare with [53-55]], fixed the already established divergences among the clades within both *R. hardwickii *and *R. muscatellum *lineages.

(v) The extension of the range of the large-sized Indian *R. microphyllum *may have appeared quite late after these events, possibly even during the Quaternary under the influence of more pronounced seasonality in the climate [53].

## Conclusion

This first genetic study dealing with the family Rhinopomatidae has enabled us to put forward phylogenetic hypotheses that differ considerably from the concepts resulting of previous morphological studies. Contrary to what was expected, we found deep allopatric genetic divergences within the *R. hardwickii *and *R. muscatellum *lineages, which suggests a separate species status for the Afro-Arabian branch of *R. hardwickii *(i.e. *R. cystops*) and for the Yemeni branch of *R. muscatellum*. In contrast, we found a surprisingly high genetic homogeneity in *R. microphyllum *(0.5% of genetic distance over 3,400 km of geographic distance). Morphological polymorphism in the genetically uniform *R. cystops *and *R. microphyllum *and the characteristics of the fossil taxon suggests plasticity of body size in this group. Considering information on the Recent and past ranges and the genealogy of the group, we expect that history of the family included (i) an early isolation in archipelago south of Western Tethyan seaway or in India, (ii) a northward- and westward spread into Mediterranean after disappearance of the marine barrier in the Late Oligocene and (iii) retreat from there after the Miocene climatic optimum.

## Methods

### Taxonomic sampling

We examined both museum specimens and individuals collected during our field excursions. The material examined for morphometric analysis (*n *= 252) covered all parts of the range (except East Asia), included nearly all nominal taxa, and included the holotypes of *R. hardwickii *Gray, 1831, *R. lepsianum *Peters, 1859, *R. kinneari *Wroughton, 1912, *R. cystops *Thomas, 1903, *R. arabium *Thomas, 1913, *R. muscatellum *Thomas, 1903, *R. seianum *Thomas, 1913, *R. pusillum *Thomas, 1920, and *R. macinnesi *Hayman, 1937. The molecular analyses were undertaken with 26 specimens representing three nominal species, namely *R. microphyllum*, *R. hardwickii*, and *R. muscatellum *(Table 2, column 1; Figure 1). Species identification followed the criteria summarized by Van Cakenberghe and De Vree [9] and Corbet and Hill [56], aided by direct comparisons with other material included in morphometric comparisons. Voucher specimens have been deposited in the collections of the National Museum, and the Department of Zoology, Faculty of Science, Charles University, both Prague, Czech Republic. Specimens were selected in order to provide a reliable geographic and taxonomic coverage of the range, and include the holotype material of *R. microphyllum*, *R. kinneari*, *R. cystops*, *R. arabium*, and *R. pusillum*. For further details concerning the specimens (including the morphometrical data), see [16,17]. Fossils reported in this paper are deposited in the collections of Department of Zoology, Charles University in Prague [see Additional file 2].

**Table 2:** Specimen and sequence information

Species – traditional	Species (clade) – proposed	Haplotype	State	Site (Region)	Collector	Accession No.
*R. hardwickii*	*R. hardwickii *(I)	IR1	Iran	Izeh (Khuzestan)	P. Benda, M. Andreas, A. Reiter, M. Uhrin	DQ337480
*R. hardwickii*	*R. hardwickii *(I)	IR1	Iran	Kuli Alireza	J. Obuch, P. Hulva	DQ337481
*R. hardwickii*	*R. hardwickii *(I)	IR2	Iran	Izeh (Khuzestan)	P. Benda, M. Andreas, A. Reiter, M. Uhrin	DQ337482
*R. hardwickii*	*R. hardwickii *(I)	IR3	Iran	Jelugir (Lorestan)	P. Benda, M. Andreas, A. Reiter, M. Uhrin	DQ337483
*R. hardwickii*	*R. cystops *(II)	LE1	Jordan	Tabaqat Fahl (Irbid)	P. Benda	DQ337484
*R. hardwickii*	*R. cystops *(II)	LE1	Jordan	Tabaqat Fahl (Irbid)	P. Benda	DQ337485
*R. hardwickii*	*R. cystops *(II)	LE1	Syria	Nimrod Fortress (Golan Heights)	P. Benda	DQ337486
*R. hardwickii*	*R. cystops *(II)	LE1	Syria	Nimrod Fortress (Golan Heights)	P. Benda	DQ337487
*R. hardwickii*	*R. cystops *(II)	YE1	Yemen	Wadi Zerig (Socotra)	V. Bejcek	DQ337488
*R. hardwickii*	*R. cystops *(II)	YE1	Yemen	Timre (Socotra)	P. Benda, A. Reiter	DQ337489
*R. hardwickii*	*R. cystops *(II)	YE1	Yemen	Wadi Zerig (Socotra)	P. Benda, A. Reiter	DQ337490
*R. hardwickii*	*R. cystops *(II)	YE1	Yemen	Old Ma'arib (Ma'arib)	P. Benda	EF443165
*R. hardwickii*	*R. cystops *(II)	YE2	Yemen	Old Ma'arib (Ma'arib)	P. Benda	EF443166
*R. hardwickii*	*R. cystops *(II)	YE3	Yemen	Al Azhlaniya (Hadramawt)	P. Benda	EF443167
*R. hardwickii*	*R. cystops *(II)	NA1	Libya	Al Jaghbub (Cyrenaica)	P. Benda, M. Andreas, V. Hanak, A. Reiter, M. Uhrin	DQ337491
*R. hardwickii*	*R. cystops *(II)	NA1	Egypt	Karnak (Qena, Upper Egypt)	P. Munclinger, P. Nova	DQ337492
*R. hardwickii*	*R. cystops *(II)	NA1	Libya	Al Jaghbub (Cyrenaica)	P. Benda, M. Andreas, V. Hanak, A. Reiter, M. Uhrin	DQ337493
*R. hardwickii*	*R. cystops *(II)	NA1	Libya	Al Jaghbub (Cyrenaica)	P. Benda, M. Andreas, V. Hanak, A. Reiter, M. Uhrin	DQ337494
*R. cf. muscatellum *	*R. sp. *(III)	YE4	Yemen	Ash-Shehir (Hardamawt)	D. Basuwayd	DQ337495
*R. cf. muscatellum *	*R. sp. *(III)	YE5	Yemen	Ash-Shehir (Hardamawt)	D. Basuwayd	DQ337496
*R. muscatellum*	*R. muscatellum *(IV)	IR4	Iran	Hormoz Isl. (Hormozgan)	P. Benda, A. Reiter	DQ337497
*R. muscatellum*	*R. muscatellum *(IV)	IR5	Iran	Izeh (Khuzestan)	P. Benda, M. Andreas, A. Reiter, M. Uhrin	DQ337498
*R. muscatellum*	*R. muscatellum *(IV)	IR5	Iran	Pir Sohrab (Baluchestan)	P. Benda, A. Reiter	DQ337499
*R. muscatellum*	*R. muscatellum *(IV)	IR6	Iran	Kahiri (Baluchestan)	P. Benda, A. Reiter, J. Obuch	DQ337500
*R. microphyllum*	*R. microphyllum *(V)	LE2	Jordan	Tabaqat Fahl (Irbid)	P. Benda	DQ337501
*R. microphyllum*	*R. microphyllum *(V)	IN1	India	Rajastan	T. Adamova	DQ337502

The present paper is the first molecular assay on taxonomy of Rhinopomatidae. All other studies on this group (as well as a preliminary routine identification of our own material) have been based on results of morphometric comparisons. Rhinopomatid taxa have been traditionally diagnosed by morphometric specificities, and distinguish these morphology-based taxa from genetic grouping by denoting them as "morphospecies".

### Molecular analysis

We extracted total genomic DNA from ethanol-preserved tissues and sequenced 402 bp of the 5'-end of the cytochrome *b *gene (according protocols published in [5]). Accession nos. [GenBank: DQ337480 – DQ337502, EF443165 – EF443167]. As multiple outgroup, we used representatives of the clade Yinpterochiroptera Teeling *et al*., 2005: *Hipposideros bicolor *[AF358131], *Rhinolophus hipposideros *[AF044660], *Rousettus leschenaulti *[AF044662] and *Epomophorus wahlbergi *[AF044642].

The sequences were aligned by eye and the dataset was processed in PAUP 4.0b10 [57]. We tested the cladistic information content and saturation level [58] by saturation tests [59]. We have inferred the model of sequence evolution in Modeltest 3.7 [60] using a hierarchical likelihood ratio test. The resulting model was used to correct distances and for maximum likelihood and Bayesian analyses. We performed distance analyses to quantify genetic gaps within the clade. We computed pairwise p-distances (Table 3, lower triangle, values used in the text to demonstrate genetic distances) and corrected distances among ingroup haplotypes. We used GTR (model fitting best our data according to Modeltest; Table 3, upper triangle) and K2P (distance used in previous studies on bat species [e.g. [23,24,61]]) corrections. The results of both corrected distance analyses were almost identical. The data were ordered by computing trees using several approaches: neighbor-joining, maximum parsimony (heuristic search with 100 random-addition sequences and the TBR branch-swapping algorithm; Figure 3a) and maximum likelihood (Figrue 3b). For the Bayesian analysis (performed in MrBayes 3.1 [62]; Figure 3c) we used GTR + I + G model, flat priors and random starting tree. We ran four chains in MCMC analysis with 10,000,000 generations and sampled trees every 100 generations. The stationary was inspected via log probability plots and the convergence diagnostics for model parameters and burn-in was used to discard first 1,000,000 generations. We repeated the Bayesian run to test for convergence. The robustness of the topologies obtained was tested by bootstrap using 1,000 replicates, and by computing posterior probabilities. We estimated the approximate dates of divergences (Figure 3d) using the linearized tree approach [63]. The difference in log-likelihoods (2 [log *L *-log *L*_clock_], [64]) of non-clock like (log *L *= -1867.36) and clock-like (log*L*_clock _= -1884.45) trees compared against χ^2 ^distribution (df = N_taxa _- 2) was not significant at the 5% level (19.50), and thus the molecular clock could not be rejected. Since there is a lack of fossils that would be useful for calibration of the tree within the family, we used a 37 Ma for *Rhinolophus-Hipposideros *split (37 Myr, [43,65]) to calibrate the tree (since the age of the fossil represents the minimum date of the fossil lineage occurrence, we provided estimates of minimum dates of divergences based on ML branch lengths). Geographic arrangements of parsimony networks were used to visualize phylogeographic pattern among haplotypes (Figure 4). We visualize the branches with minimum of mutational steps within clades revealed by tree building methods. The shortest connection between clades clades I and II and clades III and IV was displayed to indicate phylogeographic breaks within traditional *R. hardwickii *and *R. muscatellum*.

**Table 3:** P-distances (lower triangle) and corrected distances (GTR model, upper triangle) among haplotypes

species (clade)		haplotype	1	2	3	4	5	6	7	8	9	10	11	12	13	14	15
*R. hardwickii *(I)	1	IR1		0,002	0,005	0,099	0,094	0,097	0,100	0,103	0,145	0,142	0,151	0,148	0,145	0,166	0,166
	2	IR2	0,002		0,008	0,103	0,097	0,100	0,103	0,106	0,142	0,139	0,147	0,144	0,141	0,162	0,162
	3	IR3	0,005	0,007		0,093	0,093	0,091	0,099	0,102	0,143	0,140	0,148	0,145	0,142	0,163	0,163
*R. hardwickii *(II)	4	LE1	0,090	0,092	0,085		0,005	0,003	0,010	0,031	0,144	0,141	0,146	0,143	0,146	0,159	0,159
	5	YE1	0,085	0,087	0,085	0,005		0,003	0,005	0,031	0,150	0,147	0,146	0,143	0,146	0,159	0,159
	6	YE2	0,087	0,090	0,082	0,002	0,002		0,008	0,034	0,147	0,144	0,143	0,140	0,143	0,156	0,156
	7	YE3	0,090	0,092	0,090	0,010	0,005	0,007		0,036	0,149	0,146	0,152	0,150	0,152	0,166	0,166
	8	NA1	0,092	0,095	0,092	0,030	0,030	0,032	0,035		0,153	0,150	0,138	0,135	0,138	0,173	0,173
*R. muscatellum *(III)	9	YE4	0,127	0,124	0,124	0,124	0,129	0,127	0,129	0,132		0,002	0,100	0,097	0,095	0,154	0,161
	10	YE5	0,124	0,122	0,122	0,122	0,127	0,124	0,127	0,129	0,002		0,097	0,095	0,092	0,151	0,158
*R. muscatellum *(IV)	11	IR4	0,132	0,129	0,129	0,127	0,127	0,124	0,132	0,122	0,090	0,087		0,003	0,005	0,168	0,168
	12	IR5	0,129	0,127	0,127	0,124	0,124	0,122	0,129	0,119	0,087	0,085	0,002		0,003	0,165	0,165
	13	IR6	0,127	0,124	0,124	0,127	0,127	0,124	0,132	0,122	0,085	0,082	0,005	0,002		0,162	0,162
*R. microphyllum *(V)	14	LE2	0,142	0,139	0,139	0,137	0,137	0,134	0,142	0,147	0,132	0,129	0,142	0,139	0,137		0,005
	15	IN1	0,142	0,139	0,139	0,137	0,137	0,134	0,142	0,147	0,137	0,134	0,142	0,139	0,137	0,005	

## Competing interests

The author(s) declares that there are no competing interests.

## Authors' contributions

PH performed molecular and phylogenetic analyses and wrote the first version of the manuscript. IH initiated the study, discovered the fossil specimen, and contributed to the paleontological and paleobiogeographic parts of the manuscript. PB collected most of material, performed morphometric analyses, and wrote the taxonomy parts of the manuscript. All authors have read and approved the final version.

## Supplementary Material

Additional file 1 **Taxonomic structure of Rhinopomatidae**. Morphospecies vs. molecular phylogenetics. Click here for file

Additional file 2 **The late Miocene *Rhinopoma *aff. *hardwickii *from Elaiochoria, Greece**. Detailed information about the new fossil record is provided. Click here for file

Additional file 3 **Selected specimens of the fossil *Rhinopoma *aff. *hardwickii *from Elaiochoria 2, Chalkidiki, Greece compared to Recent *R. cystops***. 1: M/1-M/3, 2: P/3-M/3, 3: M/3, 4: P/4, 5: P/4, 6: M1/, 7: M2/, 8: M3/, 9: M3/, 10: C-M3/in the Recent *R. cystops *(ISZ E-62/71, Luxor, Egypt). Click here for file
