# Supplementary material for: Molecules, morphometrics and new fossils provide an integrated view of the evolutionary history of Rhinopomatidae (Mammalia: Chiroptera)
Source: BMC Evol Biol. 2007 Sep 14;7:165. doi: 10.1186/1471-2148-7-165 (PMC2249596; doi:10.1186/1471-2148-7-165)
Supplement: Additional file 1 — Taxonomic structure of Rhinopomatidae. Morphospecies vs. molecular phylogenetics. [file 1471-2148-7-165-S1.doc]

## Appendix 1: The late Miocene *Rhinopoma* aff. *hardwickii* from Elaiochoria, Greece

Here we report the first Neogene record of Rhinopomatidae. It is based on a series of 197 isolated teeth and 23 other fragments obtained from a lithified infilling of a narrow vertical karst cavity near Eleochoria, Chalkidiki, Greece (40°21’10” N, 23°09’40” E). The site is situated in the southern wall of a local quarry, some two km east of the recently reported MN13 site at Silata [1]. The lithified scree and reddish terra rossa deposit composing the infill was first sampled in 1988 (by IH), supplementary samples were also taken in 2002 and 2005. The samples were prepared with aid of acid etching and mechanical preparation of fossils and provided a relatively rich collection of isolated teeth and bone fragments of small mammals, particularly bats – almost exclusivelly just *Rhinopoma.* The other taxa were rather rare and mostly represented with single teeth only. The preliminary faunal list of the assemblage (MNI in parantheses) is as follows:

Eulipotyphla: Soricidae g.sp. indet. (1, cf. *Deinsdorfia)*

Rodentia: Sciuridae gen.et sp.indet (2, a very large form supposedly related to *Atlantoxerus*), *Csakvaromys (=Spermophilinus* see [2]) cf. *turolensis* (1), *Byzantinia* sp. (2), cf. *Rotundomys* (1), *Kowalskia* cf. *schaubi* (1), *Progonomys* sp. (5), cf. *Occitanomys* (1), cf. *Apodemus* (1),

Lagomorpha: cf. *Alilepus turolensis* (1)

Primates: cf. *Mesopithecus* sp. (1)

Carnivora: cf. *Martes* (1)

Cetartiodactyla: cf. *Lagomeryx* (1), Cervidae indet. (1, a large form)

Chiroptera: *Rhinolophus* gr. *delphinensis* (1), *Rhinopoma* aff. *hardwickii* (19), cf. *Myotis* (1, small sized)

Despite the fragmentary nature of the fossil material, the fauna indicates a Late Miocene, most probably the early Turolian age. The presence of *Progonomys,* the earliest representative of Muridae in Europe, suggests MN10 [3], the advanced morphotype in one molar resembling *Occitanomys* sp., and a large sized M^2^ supposedly belonging to *Apodemus* may suggest a younger age (MN11). *Byzantinia* (with LAD in MN 13 – [4]) similarly as other cricetids (*Kowalskia, Rotundomys*) is quite characteristic for the late Vallesian / early Turolian communities of the Eastern Mediterranean (MN 9-11) – cf. [4, 5].

Consequently, the probable stratigraphic range of the reported record of *Rhinopoma* can be narrowed to biozones MN10–MN11 (in the sense of [6]), i.e. 8–10 Ma.

*Rhinopoma* aff. *hardwickii*

*Material*: 8 mandibular fragments, 1 maxillary fragment, 9 humeral epiphyses, 5 epiphyses of radius, and 197 isolated teeth. The dental material covers 206 items: 20 C^1^, 15 P^4^, 20 M^1^, 33 M^2^, 8 M^3^, 22 C_1_, 13 P_3_, 32 P_4_, 18 M_1_, 24 M_2_, 11 M_3_.  The minimum number of individuals was estimated to be 19 (based on 33 M^2^).

*The metrical characters* (mean, n: min-max) are as follows: Maxillary: **M^1^-M^3^** =4.05, **M^1^-M^2^** =3.12, **C^1^** L 1.44 (n=19: 1.17-1.90), W=0.83 (n=4: 0.70-0.97), **P^4^** L=1.40 (n=12: 1.26-1.60), W=1.29 (n=4: 1.20-1.35), **M^1^** L=1.59 (n=13: 1.44-1.75), W1=1.69 (n=6: 1.55-1.90), W2=2.2 (n=8: 2.15-2.35), **M^2^** L=1.52 (n=17: 1.41-1.67), W1=1.92 (n=6: 1.65-2.10), W2=2.32 (n=5: 2.20-2.40), **M^3^** L=0.83 (n=8: 0.75-0.96), W=1.95 (n=6: 1.78-2.15), Mandibular: **C^alv^-M_3_** =7.50, **P_3_-M_3_** =6.8, **P_4_-M_3_** =6.12, **M_1_-M_3_** =5.00, **M_2_-M_3_** =3.30, **M_1_-M_2_** =3.46, **P_4_-M_1_** =2.85, **C_1_** L=1.11 (n=7: 0.95-1.34), W=0.69 (n=8: 0.63-0.78), **P_4_** L=1.12 (n=25: 0.98-1.26), W=0.66 (n=14: 0.60-0.76), **M_1_** L=1.50 (n=18: 1.43-1.64), tlL=0.75 (n=9: 0.70-0.82), Wtr=0.89 (n=9: 0.70-1.00), Wtl=1.08 (n=9: 1.00-1.22), **M_2_** L=1.56 (n=18: 1.35-1.67), tlL=0.82 (n=11: 0.75-0.90), Wtr=1.08 (n=11: 0.95-1.30), Wtl=1.11 (n=11: 1.00-1.22), **M_3_** L=1.39 (n=9: 1.03-1.50), tlL=0.72 (n=7: 0.68-0.77), Wtr=0.95 (n=7: 0.90-1.10), Wtl=0.6 (n=7: 0.55-0.65).

*Description:* Unfortunately in most instances only isolated teeth are available. Nevertheless, they provide a set of specificities quite sufficient at least for the generic identification of the fossil form: (1) upper canine is conspicuously narrow and long in occlusal view without a distinct cingulum, except for a cusp-like distal tapering, (2) P^4^ relativelly slender with well developed talon, long in mesio-distal respect with distinct mesio-labial tapering, (3) M^1^ and M^2^ are relatively narrow in the region of labial flexus (in sense of [7]) but conspicuous for extremely large disto-palatally projecting talons. (4) Postprotocristas are incomplete and fossas are broadly opened to talon sweep. No supplementary crests or cingular cones appear. (5) Mesostyle (or mesostylar synclinale of the plagiocrista) situated at the labial margin of the teeth. (6) Metacone region larger than paracone, a flat metastyle enlarged distally. (7) Differences between M^1^ and M^2^ in outline of a tooth and shape of talon exceed those in most other chiropteran clades. (8) M^3^ is moderately reduced, mesial third of premetacrista retained, postprotocrista complete. (9) C_1_ high and sharply pointed, nearly rounded on section with an pronounced lingual cingulum tapered in a distal secondary cusp, labial cingulum is rather indistinct (as in other unicuspids in general), (10) P_2_ narrow, broadly triangular at lateral view, almost without cingulum, (11) P_4_ oval shaped on section, with a distinct talonid-like distal margin, labial crown base broadly concave without any warping, (12) lower molars all nearly of the same size, (13) nyctalodont, (14) talonid of M_3_ narrower, its entoconid margin shifted labially (see Figure 5 for details).

*Comparisons:* We compared the fossil form with a series of extant taxa including the species of *Rhinopoma* discussed in other sections this paper and confirmed the complete correspondence to the situation in the extant genus in all morphometric traits under study. The shape and proportion of particular teeth in the fossil form seem to be almost identical with those in Recent *Rhinopoma hardwickii* (and *cystops)*. On contrary, the fossil form lacks the diagnostic features of *R. microphyllum* (i.e. high and mesially tappered palatal cingula on the upper molariform teeth, including distinct mesiopalatal cusp on P^4^, a separated hypoconal cingulum on M^2^ with a distinct cingular cusp, a more reduced M3/ with only indistinct metaconal extension of plagiocrista, haevily build lower premolars). Also in much smaller *R. muscatellum,* M^3^ is apparently more reduced (both in its total size and in that it lacks metaconal extension at all) and the mesiopalatal margin of P^4^ is more inflated (both the characters partly shared with *microphyllum*). At the same time, of course, the fossil form clearly exceeds the variation range of the Recent *R. hardwickii* (and *cystops)* in metrical characters, both in mean and maximum values.

*Comments.* There is no doubt on the generic identification of the fossil form - for all morphological traits examined, the fossil material shows the conditions that clearly distinguish Rhinopomatidae from other chiropteran families, and, moreover, it fits well with the state recorded for the Recent forms of the genus *Rhinopoma*. Nevertheless, it apparently cannot be directly coidentified with any extant species of the genus. Though there is a broad measure of correspondence between the fossil form and the Recent *Rhinopoma hardwickii* (and *cystops*) in the fine details of dental morphology, as well as in the shape of distal epiphysis of humerus and the shape of proximal epiphysis of radius and other characters, the fossil form seems to exceed the range of variation in the Recent *R. hardwickii*, both in mean values and in the highest values (comp. Figure 2a). The fossil material shows relatively broad size variation, however still it seems unlikely that it would include more than one species; unfortunately, in the moment, we feel unable to answer that question for sure. The material is still too fragmentary for a detailed search on the source of the variation. For the same reason we do not try to put any definite species identification of the fossil form nor do we propose a new species name for it. Until supplementary material will appear we keep it under a provisional name *Rhinopoma* aff. *hardwickii.*

## References

1. Vasileiadou KV, Koufos GD, Syrides GE: **Silata, a new locality with micromammals from the Miocene/Pliocene boundary of the Chalkidiki peninsula, Macedonia, Greece**. Deinsea 2003, **10:**549–562.
2. Kretzoi M, Fejfar O: **Sciurids and Cricetids (Mammalia, Rodentia) from Rudabánya**. Palaeontographia Italica 2004, **90**:113–148.
3. De Bruijn H., van DAm JA, Daxner-Hock G, Fahlbusch V, Storch G: **The Genera of the Murinae, Endemic Insular Forms Expected, of Europe and Anatolia During the Late Miocene and Early Pliocene.** In *The Evolution of Western Eurasian Neogene Mammal Faunas*. Edited by Bernor RL, Fahlbusch V, Mittmann H-W. New York: Columbia University Press, 1996:253–260.
4. Koufos GD: **Late Miocene mammal events and biostratigraphy in the Eastern Mediterranean.** Deinsea 2003, **10**:343–371.
5. Unay E, de Bruijn H, Sarac G: **The Oligocene rodent record of Anatolia: a review.** Deinsea 2003, **10:**531–538.
6. Mein P: **Updating of MN zones**. In *European Neogene Mammal Chronology*. Edited by Lindsay EH, Fahlbusch V, Mein P. New York: Plenum Press; 1990:73–90.
7. Menu H: **Morphotypes dentaires actuels et fossiles des chiroptéres vespertilioninés. Ie partie: Etude des morphologies dentaires**. Palaeovertebrata 1985, **15**:71–128.
