# Supplementary figures and images for: Molecules, morphometrics and new fossils provide an integrated view of the evolutionary history of Rhinopomatidae (Mammalia: Chiroptera)
Source: BMC Evol Biol. 2007 Sep 14;7:165. doi: 10.1186/1471-2148-7-165 (PMC2249596; doi:10.1186/1471-2148-7-165)

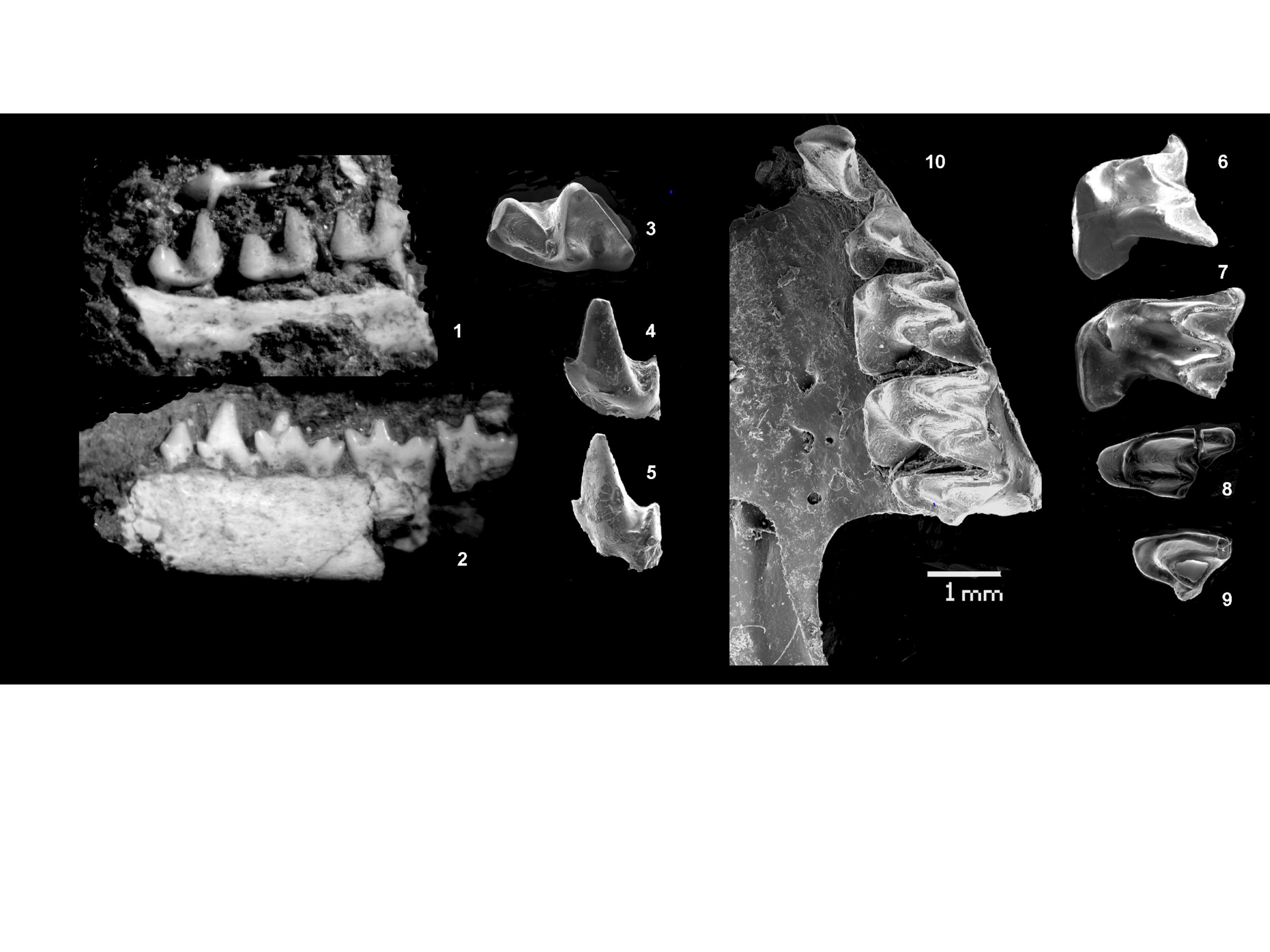

Supplement: Additional file 2 — The late Miocene Rhinopoma aff. hardwickii from Elaiochoria, Greece. Detailed information about the new fossil record is provided. [file 1471-2148-7-165-S2.png]
