# Supplementary material for: Molecules, morphometrics and new fossils provide an integrated view of the evolutionary history of Rhinopomatidae (Mammalia: Chiroptera)
Source: BMC Evol Biol. 2007 Sep 14;7:165. doi: 10.1186/1471-2148-7-165 (PMC2249596; doi:10.1186/1471-2148-7-165)
Supplement: Additional file 3 — Selected specimens of the fossil Rhinopoma aff. hardwickii from Elaiochoria 2, Chalkidiki, Greece compared to Recent R. cystops. 1: M/1-M/3, 2: P/3-M/3, 3: M/3, 4: P/4, 5: P/4, 6: M1/, 7: M2/, 8: M3/, 9: M3/, 10: C-M3/in the Recent R. cystops (ISZ E-62/71, Luxor, Egypt). [file 1471-2148-7-165-S3.doc]

## Appendix 2:

## Taxonomic structure of Rhinopomatidae: morphospecies vs. molecular phylogenetics

The earliest synopses identified only one species within the family and genus, *R. microphyllum*, characterized with a very wide morphometric variation [1, 2]. Thomas [3] first demonstrated a marked phenotypic diversity within the group and distinguished five species, viz. *microphyllum*, *hardwickii*, *cystops*, *sumatrae*, and *muscatellum*, the latter three he described as new species. Later, Thomas [4, 5] supplemented the comparisons with further material and depicted three other forms: *R. cystops arabium*, *R. muscatellum seianum*, and *R. pusillum*. Wroughton [6] also described a new separate species, *R. kinneari*, from India, and Hayman [7] described an additional subspecies, *R. cystops macinnesi*, from Kenya.

Ellerman and Morrison-Scott [8] reconsidered all these proposals and arranged the Palaearctic and Oriental named taxa into three species: Afro-Arabian *R. microphyllum* (Brünnich, 1782), Indian *R. kinneari* Wroughton, 1912 (both large-sized and monotypic), and a small-sized polytypic species *R. hardwickii* Gray, 1831, distributed over a wide geographic range from West Africa to Thailand, with six subspecies (*hardwickii*, *cystops*, *muscatellum*, *arabium*, *seianum*, and *pusillum*). Later, Felten [9] demonstrated extensive similarity between *microphyllum* and *kinneari* and suggested that they should be regarded as a single species (under the former name). Kock [10], who re-examined mouse-tailed bats from the Afro-Arabian part of the range, recognized there two species (conforming to [8]) with several subspecies: *R. m. microphyllum* and *R. m. tropicalis* (newly described), and *R. h. cystops*, *R. h. muscatellum*, *R. h. seianum*, *R. h. macinnesi*, and *R. h. senaariense* (in addition to the Asiatic *R. m.* *kinneari*, *R. m*. *sumatrae*, and *R. h. hardwickii*). DeBlase et al. [11] demonstrated that two small-sized forms occur in sympatry in southwest Asia, *R. h. hardwickii* and *R. h. muscatellum* (including *seianum* and *pusillum*), and proposed a separate species status of them. Schlitter and DeBlase [12] recognized a new form of *R. microphyllum* in southern Iran, and described it as *R. m. harrisoni*.

A profound survey of the group was undertaken by Hill [13] who revised all types, reexamined the diagnostic characters and their variation in the particular forms and concluded that the genus *Rhinopoma* consists of three species and 11 subspecies: *R. microphyllum* (with five subspecies), *R. hardwickii* (four subspecies), and *R. muscatellum* (two subspecies). Later, Nader and Kock [14] described a new subspecies from southwestern Saudi Arabia, *R. microphyllum* *asirensis,* and Khajuria [15] described another new form from western Iraq, as a separate species *R. hadithaensis*. Except for the latter, and with a few minor adjustments, the above content (three species and 11 subspecies) represents the standard concept of the genus as established by Koopman [16, 17].

The essential analysis of the topics was undertaken by van Cakenberghe and de Vree [18] who compared very extensive material involving ca. 1150 specimens of all named taxa of the family Rhinopomatidae (except for the form described by Khajuria [15]). They concluded that the genus consists of four species with a number of subspecies (including one newly described): *R. microphyllum* with *R. m. microphyllum* (Brünnich, 1782) (African part of the range, plus N Arabia, Iran, Afghanistan, and Pakistan; including *lepsianum* Peters, 1859, *cordofanicum* Heuglin, 1877, *tropicalis* Kock, 1969, and *harrisoni* Schlitter and DeBlase, 1974), *R. m. asirensis* Nader and Kock, 1982 (SW Saudi Arabia), *R. m. kinneari* Wroughton, 1912 (India), and *R. m. sumatrae* Thomas, 1903 (Sumatra); *R. hardwickii*, with *R. h. hardwickii* Gray, 1831 (India, Pakistan, and Afghanistan), *R. h. arabium* Thomas, 1913 (African, Arabian, and Persian parts of the range, with the exception of central Sahara; including *sennaariense* Kock, 1969), *R. h. cystops* Thomas, 1903 (central Sahara in Egypt, Niger, and Algeria), and *R. h. sondaicum* van Cakenberghe and de Vree, 1994 (Sunda Archipelago), *R. muscatellum*, with *R. m. muscatellum* Thomas, 1903 (Persian Gulf countries, including *pusillum* Thomas 1920) and *R. m. seianum* Thomas, 1913 (eastern Iran, Afghanistan, Pakistan, and India), and monotypic *R. macinnesi* Hayman, 1937 (Kenya, Ethiopia, Somalia, and Eritrea). In addition, Kock et al. [19] reported *R. muscatellum* from Yemen (which extended the distribution range of the species far to the west) and demonstrated that the name *R. hadithaensis* Khajuria, 1988 is a junior synonym of *R. microphyllum*. For a review of the current taxonomy of Rhinopomatidae based on the above-mentioned records, see [20].

Our material comes from the center of the family range (i.e. from North Africa, Arabia, Iran and India), samples from the marginal regions were unfortunately not at our disposal for genetic analyses. It is worth mentioning, in these connections, that the records of *Rhinopoma* east of India (Thailand, Sunda Islands) are restricted to few historical cases and were not repeated during last hundred years. This holds both for *microphyllum* (reported from northern Sumatra) and *hardwickii* (reported from Krabi, southern Thailand and from Sunda Islands without any specifications – comp. [21]).

In agreement to results of previous studies [13, 18] we confirmed that *R. hardwickii* from Upper Egypt differs clearly from the population from Arabia, SE Mediterranean and North Africa (outside of the Sahara) in morphometrical characters. Since Thomas [4] it is widely accepted that the two allopatric forms represent different taxonomic entities, i.e. subspecies, *R. h. cystops* (central Sahara), and *R. h. arabium* (North Africa except for Central Sahara, to the Middle East). The extremely large body size found in Libyan sample led us [22] to conclusion that it prove a geographic variation in body size within *arabium,* corresponding to the later identified genetic dissimilarities between the Levantine and Libyan forms (3%). Nevertheless, the mtDNA haplotypes from Libya are identical with the haplotype from Upper Egypt, i.e. those representing topotypes of *cystops*, the form exhibiting the smallest body size throughout whole distribution range. Consequently, the traditional proposal on taxonomic significance of the metrical differences between Arabian and Upper Egypt populations and subspecific arrangement based on them [4, 13, 18,20, 22] should be rejected. The molecular data suggest that the Afro-Arabian populations formerly arranged in morphospecies *R.hardwickii* (i.e. the clade II in this paper) split into two groups, possibly subspecies, ranged in Arabia and in Africa. The prior name available for the former one is *arabium* Thomas, 1913 (type locality Wasil, W Yemen), and *cystops* Thomas, 1903 (type locality Luxor, Egypt) for the latter one, which provides at the same time the prior name for the clade II, to which both the taxa belong.

The Iran populations of *R. hardwickii* have been considered as belonging to subspecies *R. h. arabium* because of the lack of significant difference from the Levantine and Arabian populations in morphometric traits, and apparent differences from the Indian form [18, 22, 23]. However, the present paper reveals that the Iran populations represent a distinct clade (I) within *R. hardwickii*, separated by genetic distance of about 8–9% from the Afro-Arabian clade II (i.e. *cystops*). In regard to the genetic species concept (discussed above) it seems reasonable to consider the clades I and II as separate species. Tentatively, for the geographic reasons, we coidentify the Iran one (I) with *Rhinopoma hardwickii* Gray, 1831 that was described from Bengal, India. The morphological differences between Iran population and the Indian, Afghani, and Pakistani populations [13, 18] suggest a different taxonomic status of them (that should be formally emphasized with a new name for the Iran form).

Of course, without more detailed coverage of the range, and the detailed data on the genetic structure and variation patterns of the topotypic populations of *R.hardwickii* s.str. we are unable to solve the taxonomic problems of that species.

Neverheless, even now it is possible to conclude that name *R. hardwickii* should not be applied to the populations belonging to the clade II (i.e. the *R. hardwickii* populations of North Africa and Arabia, incl. the Levant, sensu Simmons [20]). The prior name for them is *Rhinopoma cystops* Thomas, 1903, nomenclatoric status of its subspecies would be then: *R. cystops cystops* Thomas, 1903 (Africa), and *R. cystops arabium* Thomas, 1913 (Arabia and the Levant), exactly according to the original proposal by Thomas [4].

Set of the samples obtained from morphospecies *R. muscatellum* split into two distinct phylogroups (clades III and IV) separated with 8–9% of genetic distance. Genetic isolation, allopatric distribution and internal homogeneity of both clusters should justify their species status. For the geographic and morphological proximities to the type series of (type locality Wadi Bani Ruha, Oman, i.e. some 400 km from the Island of Hormoz), we tentatively refer clade IV (Iran, from Hormoz to Baluchistan) to *R. muscatellum* s.str. No name is available for the Yemeni form (clade III), here is provisionally named *Rhinopoma* sp.n.

The unexpectedly small genetic distance between representatives of the nominative forms *R. microphyllum microphyllum* from the Levant and *R. m. kinneari* from western India (0.5%) excludes their separate species status (comp. e.g. [8]) and does not support even a separate subspecific status of them either. The morphological differences were declared in pelage coloration and body size, as the Indian form was described to be slightly larger [13, 9, 21]. Relevance of the diagnostic criteria was doubted, however, by Kock [10] and also van Cakenberghe and de Vree [18] or Hill [13], who suggested possible synonymy of *kinneari* and *microphyllum*. The status of the forms most distant from them in geographical and morphological points, i.e. *sumatrae* and *asirensis* (see [14]) remains for obvious reason (see above) an open question.

## References

1. Dobson GE: *Catalogue of the Chiroptera in the Collection of the British Museum*. London: British Museum; 1878.
2. Anderson J: *Zoology of Egypt: Mammalia. Revised and completed by W. E. de Winton, F.Z.S*. London: Hugh Rees Ltd; 1902.
3. Thomas O: **On the species of the genus *Rhinopoma***. Ann Mag Natur Hist 1903, (**7**)**11:**496–499.
4. Thomas O: **Some new Fer from Asia and Africa**. Ann Mag Natur Hist 1913, (**8**)**12:**88–92. 28.
5. Thomas O: **Scientific results from the Mammal Survey**. **No**. **XXII**. **A**. **A new bat of the genus *Rhinopoma* from S**. **E**. **Persia**. J Bombay Natur Hist Soc 1920, **27:**25.
6. Wroughton RC: **Some new Indian mammals**. J Bombay Natur Hist Soc 1912, **21**:767–773.
7. Hayman RW: **Postscript**. In: *Mammals collected by the Lake Rudolph Rift Valley Expedition, 1934*. Edited by St. Leger J. Ann Mag Natur Hist 1937, (**10**)**19:**530–531.
8. Ellerman JR, Morrison-Scott TCS: *Checklist of the Palaearctic and Indian Mammals 1758 to 1946*. London: British Museum (Natural History); 1951.
9. Felten H: **Bemerkungen zu Fledermäusen der Gattungen *Rhinopoma* und *Taphozous*** (**Mammalia, Chiroptera**). Senckenberg Biol 1962, **43:**171–176.
10. Kock D: **Die Fledermaus-Fauna des Sudan** (**Mammalia, Chiroptera**). Abh Senckenberg Naturforsch Ges 1969, **521:**1–238.
11. DeBlase AF, Schlitter DA, Neuhauser HN: **Taxonomic status of *Rhinopoma muscatellum* Thomas** (**Chiroptera**: **Rhinopomatidae**) **from Southwest Asia**. J Mammal 1973, **54:**831–841.
12. Schlitter DA, DeBlase AF: **Taxonomy and geographic distribution of *Rhinopoma microphyllum*** (**Chiroptera**: **Rhinopomatidae**) **in Iran, with the description of a new subspecies**. Mammalia 1974, **38:**657–665.
13. Hill JE: **A review of the Rhinopomatidae** (**Mammalia**: **Chiroptera**). Bull Brit Mus Natur Hist Zool 1977, **32:**29–43.
14. Nader IA, Kock D: ***Rhinopoma microphyllum asirensis* n**. **subsp**. **from southwestern Saudi Arabia** (**Mammalia**: **Chiroptera**: **Rhinopomatidae**). Senckenberg Biol 1983, **63:**147–152.
15. Khajuria H: **A new species of rat-tailed bats** (**Chiroptera**: **Rhinopomatidae**) **from Iraq**. Rec Zool Surv India 1988, **85:**391–402.
16. Koopman KF: **Order Chiroptera**. In *Mammal Species of the World. A Taxonomic and Geographic Reference*. Edited by Wilson DE, Reeder DM. Washington and London: Smithsonian Institution Press; 1993**:**137–241.
17. Koopman KF: *Chiroptera: Systematics. Handbook of Zoology. Volume VIII. Mammalia*. Berlin and New York: Walter de Gruyter; 1994.
18. Van Cakenberghe V, De Vree F: **A revision of the Rhinopomatidae Dobson 1872, with the description of a new subspecies** (**Mammalia**: **Chiroptera**). Senckenberg Biol 1994, **73:**1–24.
19. Kock D, Al-Jumaily M, Nasher AK: **On the genus *Rhinopoma* E**. **Geoffroy 1818, and a record of *Rh***. ***muscatellum* Thomas 1903 from Yemen** (**Mammalia, Chiroptera, Rhinopomatidae**). Senckenberg Biol 2001, **81:**285–287.
20. Simmons NB: **Order Chiroptera**. In *Mammal Species of the World: A Taxonomic and Geographic Reference. Third Edition*. *Volume 1*. Edited by Wilson DE, Reeder DM. Baltimore: The John Hopkins Univ Press; 2005:312–529.
21. Corbet GB, Hill JE: *The Mammals of the Indomalayan Region: a Systematic Review*. London: Oxford Univ Press; 1992.
22. Benda P, Hanak V, Andreas M, Reiter A, Uhrin M: **Two new species of bats** (**Chiroptera**) **for the fauna of Libya**: ***Rhinopoma hardwickii* and *Pipistrellus rueppellii***. Myotis 2004, **41–42:**109–124.
23. DeBlase AF: **The bats of Iran**: **systematics, distribution, ecology**. Field Zool (NS) 1980, **4:**1–424.
